# Plant Biosensors Analysis for Monitoring Nectarine Water Status

**DOI:** 10.3390/bios14120583

**Published:** 2024-11-30

**Authors:** María R. Conesa, Wenceslao Conejero, Juan Vera, M. Carmen Ruiz Sánchez

**Affiliations:** Irrigation Department, Centro de Edafología y Biología Aplicada del Segura (CEBAS-CSIC), P.O. Box 164, 30100 Murcia, Spain; wenceslao@cebas.csic.es (W.C.); jvera@cebas.csic.es (J.V.); mcruiz@cebas.csic.es (M.C.R.S.)

**Keywords:** digitalised irrigation systems (DISs), LVDT sensors, management allowed deficit (MAD), microtensiometers (MTs), time-domain reflectometry (TDR), plant water relations

## Abstract

The real-time monitoring of plant water status is an important issue for digital irrigation to increase water productivity. This work focused on a comparison of three biosensors that continuously evaluate plant water status: trunk microtensiometers (MTs), trunk time-domain reflectometry (TDR), and LVDT sensors. During the summer and autumn seasons (DOY 150–300), nectarine trees were subjected to four different consecutive irrigation periods based on the soil Management Allowed Deficit (MAD) concept, namely: MAD_10_ (light deficit); MAD_50_ (moderate deficit); MAD_100_ (severe deficit), and MAD_0_ (full irrigation). Measurements of stem water potential (Ψ_stem_) and leaf gas exchange were recorded on representative days. A continuous measurement of the plant water status of Ψ_trunk,_ MDS, and K_trunk_ revealed the water deficits imposed on the soil. The highest water deficit observed at the end of the MAD_100_ period (Ψ_stem_ = −2.04 MPa and Ɵ_v_ = 17%) resulted in a minimum value of Ψ_trunk_ (−1.81 MPa). The maximum value of MDS (408 µm) was observed earlier than that of Ψ_trunk,_ motivated by the low sensitivity of MDS at Ψ_trunk_ < −1.2 MPa and Ψ_stem_ < −1.5 MPa due to a decrease in the tissue elasticity of the trunk when severe water deficit conditions are reached. Both Ψ_trunk_ and Ψ_stem_ were more dependent on soil water content, while MDS was more responsive to environmental changes. K_trunk_ was the weakest indicator for determining plant water status, although when expressed as a daily fraction of depletion (K_trunk_FD), it improved, evidencing a process of hysteresis. Ψ_trunk_ showed the highest sensitivity, suggesting the potential use of MTs as a valuable biosensor for monitoring nectarine water status in digital agrosystems.

## 1. Introduction

The incorporation of new technologies that link data-driven decisions with efficient irrigation management is essential for the digitalisation of Mediterranean agriculture [1]. The term digital farming is used to describe the use of web-based data platforms and the Internet of Things (IoT) to enable fields to be connected to Wireless Sensor Networks (WSNs) through the use of autonomous equipment that monitors, stores, and communicates data [2,3,4]. In this sense, plant sensing is the ultimate achievement in providing farmers with sufficient tools to increase yields while minimising environmental impact [5,6].

Digitalised irrigation systems (DISs) prevent over and under irrigation, which can cause water stress in plants and reduce productivity, by ensuring that crops receive the exact amount of water they need [1,3,5,6]. Through DIS, the irrigation system is automated by acting on solenoid valves to trigger/stop irrigation with real-time information from the soil–plant–atmosphere water continuum without any manual intervention [7,8,9]. In this sense, DIS is performed through remote sensing, drones and satellites [4] or ground sensors connected by robust transmission units (including transducers, microcontrollers, batteries, etc.) capable of storing data and transferring protocols through bidirectional WSN [1,4].

Continuous measurements of volumetric soil water content (Ɵ_v_) using real-time feedback protocols for automated irrigation have achieved promising results in recent years [6,7,10,11]. Automated soil-based irrigation is commonly performed using field capacity (Ɵ_FC_) or Management Allowed Depletion (Ɵ_MAD_) values, defined as a percentage of available water. The concept of MAD represents the total water available to the crop so that it does not experience significant plant water stress affecting crop productivity [12,13]. In previous experiences in nectarine trees, the Ɵ_MAD_ approach has been widely used as a suitable criterion to trigger irrigation whenever Ɵ_v_ decreases to the established target value [14,15,16].

Accurate irrigation also requires plant-based measurements that can be easily automated and interpreted by fruit growers [17]. Although current applications of automated plant-based methods are still under development, the advantage of plant measurements for irrigation management is based on the use of the plant as a biosensor to assess the water status of the plant, as they represent the interphase between soil and atmosphere, as well as including the physiological response of the plant to available water [18,19].

Stem water potential at midday (Ψ_stem_) is considered the most reliable plant indicator to determine the level of water stress in fruit orchards [14,17,20,21,22]. Its practical measurement in the field requires the use of a pressure chamber, covering the leaves to stop transpiration [23] for at least 1 h before its measurements [24]. All this means a temporally destructive and non-automated measurement [17].

Recently, new biosensors known as microtensiometers (MTs) have emerged as a non-destructive method to monitor plant water status continuously. MTs are based on a microelectromechanical pressure sensor that is embedded directly (minimally invasive) into the tree trunk, providing continuous measurements of the trunk water potential (Ψ_trunk_). The operating principle of tensiometry is based on the equilibrium between a sample and an internal volume of liquid water via a vapour gap and a microporous ceramic membrane so that, by means of the pressure differences between the vapour and the internal liquid, the water potential can be measured [25]. They can operate reliably below −10 MPa, with response times of approximately 15 min providing readings in the same units as the traditional pressure chamber method [26]. Ψ_trunk_ measurements involve the complete water pathway from the roots, which absorb the water available in the soil, to the stem, whereby water is transported through the xylem to fruits and leaves, where it evaporates into the air (transpiration via stomata) [27]. MTs are able to directly monitor the xylem water potential, determining the driving force for water transport between the bark and the xylem vessels [28]. Recently, the use of MTs to continuously monitor Ψ_trunk_ has been successfully validated under different environmental and irrigation conditions with promising results in vines [29], cotton [30], nectarine [15], pears [26,31], and apple [27,32] trees. This fact has led to significant research interest in the evaluation of these MTs as biosensors for efficient irrigation management.

Another minimally invasive method is the measurement of trunk dielectric permittivity (K_trunk_) using TDR sensors (*Time Domain Reflectometry*) [33,34]. The equation developed by Topp et al. [35] established the principles relating to K and Ɵ_v_ in the soil based on the high value of K in water (80), compared to that of other elements such as air (1) and the mineral and organic fractions (between 3 and 7) present in the soil. Since K_trunk_ is highly dependent on water content, measuring K_trunk_ could help to determine the moisture content in a single tree trunk. The TDR sends an electromagnetic pulse through the trunk and measures the reflected signals to determine the K_trunk_ in a porous material such as wood [36,37]. TDR rods penetrate directly into the sapwood, capture daily and seasonal changes in K_trunk_, and ultimately determine fluctuations in tree trunk hydration [38].

Based on the knowledge that plant growth is a valuable indicator of water stress, trunk diameter fluctuations (TDFs) provided using *Linear Variable Differential Transformer* (LVDT) sensors have also been proposed as continuous parameters of plant water status for irrigation scheduling [39]. During the night, the stem rehydrates and its diameter reaches its maximum near sunrise. The trunk diameter decreases throughout the day until it reaches its minimum diameter a few hours after solar noon when evaporative demand is at its maximum [40]. The difference between the maximum and minimum trunk diameters over a 24-h period is known as maximum daily trunk shrinkage (MDS), which has been well-correlated with discrete Ψ_stem_ determinations in many fruit tree species [27,41].

All these biosensors, MTs, TDR, and LVDT, as well as others (i.e., leaf turgor, cavitation or sap flow sensors), can be used to provide a comprehensive understanding of plant water status and infer growth patterns. For example, the TDR-trunk and MTs sensors could indicate a period of water deficit and the intensity of plant water stress supported by the registered values of K_trunk_ and Ψ_trunk_, while the LVDT sensor gives information corresponding to variations in trunk diameter due to plant water loss. Blanco and Kalcits [31] addressed the comparison of MDS and Ψ_trunk_ in pear trees under controlled deficit conditions. The authors explained that although MDS detected water stress earlier than Ψ_trunk_, MDS did not increase at the same rate, showing a delay in tree trunk hydration changes. The same authors demonstrated the suitability of Ψ_trunk_ as a reliable plant water status indicator in apple trees with high sensitivity and less variability than sap flow measurements, MDS, and the differential temperature between the canopy and the air [27].

Furthermore, two recent papers have successfully demonstrated the suitability of continuous measurements of Ψ_trunk_ [15], and K_trunk_ [16] to assess the impact of automated MAD-based irrigation scheduling in nectarine trees. However, the comparison between continuous measurements of K_trunk_, Ψ_trunk_, MDS and other conventional plant water status indicators in response to different soil water deficits has not been reported so far.

Therefore, the main objective of this study was to compare the use of MTs, TDR, and LVDT plant biosensors to measure plant water status in field-grown adult nectarine trees under different irrigation regimes. We hypothesised that as Ψ_trunk_ values are more influenced by water deficits, they can sense the plant water stress earlier than K_trunk_ and MDS. In addition, a higher dependence between Ψ_trunk_ vs. K_trunk_ and MDS indexes is expected. In this study, irrigation was automatically managed by real-time Ɵ_v_ values yielding different soil water deficit situations. The sensitivity of Ψ_trunk_, K_trunk_, and MDS measurements to determine the water deficit of nectarine trees is also discussed.

## 2. Materials and Methods

### 2.1. Description of the Experimental Orchard

The present study was carried out during the northern hemisphere summer–autumn period from June to October 2022 (DOY, day of the year, 150–300), spanning an experimental period of 190 days. The selected orchard of 0.5-ha corresponded to early maturing (harvested in early May) adult nectarine trees (*Prunus persica* (L.) Batsch, cv. Flariba grafted onto GxN-15 rootstock), planted in 2010 at a tree spacing 6.5 m × 3.5 m, trained to an open-centre canopy, and located at the CEBAS-CSIC experimental field-station in Santomera, Murcia, Spain (38° 06′ 31″ N, 1° 02′ 14″ W, 110 m altitude).

The soil profile in the layer corresponding to the main active root zone (0–0.50 m) was stony with a clay loam texture (clay fraction: 41% illite, 17% smectite, and 30% palygorskite) and had an average bulk density of 1.43 g cm^−3^, calcium carbonate content of 45%, and organic matter content of 1.4%. Soil water content (θ_v_) data at field capacity (FC) and wilting point (WP) amounted to 0.29 and 0.14 m^3^ m^−3^, respectively, as obtained from the textural data [42].

In the experiment, trees were drip-irrigated with a single line per tree row, with four self-pressure compensated emitters (4 L h^−1^ each) per tree located at 0.50 and 1.30 m on both sides of the tree trunk. Irrigation was automatically managed by soil water content threshold values, as described below (see Section 2.2).

The irrigation water, with an electrical conductivity (EC_25 °C_) of 0.8 dS m^−1^ and pH of 7, came from the water distribution network of the Irrigation Community of Santomera ‘Azarbe del Merancho’. The phytosanitary treatments together with the standard cultural practices (i.e., pruning, manual weed control, and fruit-thinning, among others) were carried out by the technical staff of the CEBAS-CSIC experimental station following local fruit growing practices.

Within the experimental station (250 m from the nectarine orchard), an automated weather station continuously monitored the main agrometeorological variables: air temperature (T_air_), relative humidity (RH), solar radiation, wind speed, crop reference evapotranspiration (ET_0_), [13] and rainfall. The daily vapour pressure deficit was calculated from the daily minimum RH and maximum T_air_ values, while the air–water potential (Ψ_air_) was calculated using the Nobel equation [43].

### 2.2. Soil Measurements

Volumetric soil water content (Ɵ_v_, %) data were obtained from multidepth EnviroScan^®^ capacitance probes (Sentek Sensor technologies, Sidney, Australia) installed in PVC access tubes located 0.1 m from the emitter located close (0.5 m) to the tree trunk on four representative nectarine trees (*n* = 4). Each capacitance probe had sensors at 0.10, 0.30, 0.50, and 0.70 m depth, and was connected to a radio transmission unit. The probes were normalised and calibrated for clay-loam soil [44].

Soil water potential (Ψ_soil_, kPa) was estimated using granular matrix sensors (WEENAT, Nantes, France) installed in the wet bulb of two nectarine trees (*n* = 2) at a soil depth of 0.30 and 0.60 m. Data were recorded and visualised on the manufacturer’s cloud platform (www.weenat.com accessed on 27 November 2024).

### 2.3. Soil Water Deficit Protocols

Four irrigation regimes were consecutively applied to obtain different soil water deficit conditions (from Ɵ_v_ values at 0–0.50 m depth), based on the Maximum Allowed Deficit (MAD) [12,13], as follows:

(a) Light water deficit: MAD at 10% (DOY, days of the year, 150–180).
(b) Moderate water deficit: MAD at 50% (DOY 181–210).
(c) Severe water deficit: MAD at 100%, means non-irrigation (DOY 211–245).
(d) Full irrigation: MAD at 0% when Ψ_stem_ in plants reached −2.0 MPa (DOY 246–300).

A telemetry system (ADCON Telemetry) was used to monitor Ɵ_v_ and automate the irrigation algorithms. The transmission units sent the data via radio to a gateway which was connected to a web server (addVANTAGE Pro 6.6) for data recording, processing, visualisation, and acting on latched solenoid valves. The amount of irrigation water applied was measured with flowmeters located at the beginning of the plot piping and connected to the telemetry system.

### 2.4. Plant Measurements

Regarding **temporal** plant water status determinations, the seasonal dynamics of midday stem water potential (Ψ_stem_) and leaf gas exchange were obtained every 7 to 10 days on clear days.

Ψ_stem_ (MPa) was measured using a pressure chamber (Soil Moisture Equip. Crop. Model 3000, Santa Barbara, CA, USA) on mature leaves located on the shaded side of the tree and close to the tree trunk around midday (13:00–14:00 h, GMT+2). The leaves were covered with foil zip-lock bags for at least 2 h before the measurements. One leaf per tree was cut from four trees (*n* = 4) and immediately placed in the chamber following the recommendations of McCutchan and Shackel [45].

On the same trees, net photosynthesis (P_n_, µmol m^−2^ s^−1^), stomatal conductance (g_s_, mmol m^−2^ s^−1^), and leaf transpiration (E, µmol mmol^−1^) were measured on one mature sun-exposed leaf per tree (*n* = 4) in the early morning (9:00–10:00 h, GMT+2) using a portable gas exchange system (LI-COR, LI-6400) at an ambient photon flux density (PPFD) ≈ 1200–1500 μmol m^−2^ s^−1^ and CO_2_ concentration ≈ 400 μmol mol^−1^. In addition, the transpiration efficiency (WUE_T_) was calculated as the ratio P_n_/E (µmol mmol^−1^).

**Figure 1:**
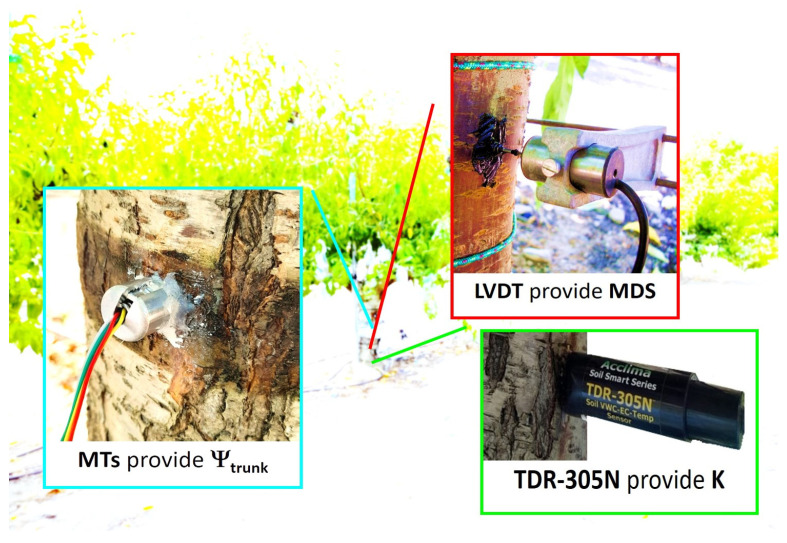
Details of plant biosensors (MTs, TDR-305N, and LVDT) installed on a nectarine tree trunk.

Regarding **continuous** plant water status determinations, the seasonal dynamics of trunk water potential (Ψ_trunk_), dielectric permittivity (K_trunk_), and diameter fluctuations were monitored using biosensors installed on the north-shaded side of four nectarine trees (*n* = 4), away from direct sunlight (Figure 1). In all cases, the 15-min processed data were transmitted via SDI-12 protocol using the same telemetry network used for θ_v_.

Trunk water potential (Ψ_trunk_, MPa) was monitored using microtensiometers (MTs; FloraPulse, Davis, CA, USA), consisting of a tensiometer microchip that is embedded directly into the woody tissue of the tree. The insertion of the metal support of the MTs requires a drilled hole followed by the application of kaolin to allow water flow and fix the biosensor [25,46].

The trunk dielectric permittivity (K_trunk_) was monitored with TDR-305 N (Acclima Inc., Meridian, ID, USA) sensors, which were installed closer to the MTs following the recommendations described in Schwartz et al. [47]. The TDR-305 N sensor consists of three 0.05 m long waveguides. More details on the installation can be found in Conesa et al. [16]. In addition, the fraction depletion of K_trunk_ (K_trunk_FD, %) was calculated daily as:

K_trunk_FD (%): (K_max_ − K′)/(K_max −_ K_min_) (1)

where K_max_ is the maximum K value at MAD_0_, K_min_ is the minimum K value at MAD_100_, and K′ is the mean diurnal K value.

Both the MTs and the TDR-305N sensors were placed 0.40 m above the soil surface and insulated with a thermal blanket following the recommendations of Saito et al. [48].

Trunk diameter fluctuations (TDFs, μm) were monitored with linear variable displacement transducers (LVDTs, Solartron Metrology, Bognor Regis, UK, model DF ± 2.5 mm, precision ± 10 μm) mounted on aluminium and invar holders (64% Fe and 35% Ni), yielding minimal thermal expansion. From these, the maximum daily shrinkage (MDS) was calculated as the daily difference between the maximum and the minimum diameter [39]. Similarly, the daily range (DR) of the Ψ_trunk_ and K_trunk_ values was also calculated as the difference between the maximum and minimum values recorded using the MTs’ time spam (Ψ_trunk_ DR) and TDR-305N (K_trunk_DR).

Daylight courses of Ψ_stem_ and leaf gas exchange parameters were carried out at the end of each irrigation period: light deficit (DOY 174), moderate deficit (DOY 210), severe deficit (DOY 244), and full irrigation (DOY 280). On these dates, Ψ_stem_ and leaf gas exchange were measured hourly on one leaf per tree in each replicate (*n* = 4).

### 2.5. Sensitivity Analysis

For all plant-based water status indicators, the signal intensity (SI) was calculated as the ratio of all data registered in the periods of severe water deficit (MAD_100_) and full irrigation (MAD_0_). To determine noise, the coefficient of variation (CV) of the measurements was calculated for each indicator. The sensitivity of the indicators was determined using two algorithms:

- Traditional method S = SI/CV [39]. The higher the S value, the higher the sensitivity.
- Corrected method S* = (SI-1)/CV [49]. S* = 0: indicates the absence of sensitivity to water deficits; S* > 1: indicates sensitivity to water deficits. If S* is <0, it indicates the presence of anomalous values for the plant indicator.

It should be noted that comparisons between discrete and continuous data on plant water status were made using the same time interval of measurements. Thus, the day of sampling and the type and size of samples did not interfere with the analysis.

### 2.6. Experimental Design and Data Analysis

The nectarine trees were arranged in the orchard following a completely randomised experimental design with four replications, each consisting of a row of six trees, the central ones were used for measurements, and the remaining served as border trees. 

Statistical comparisons were considered significant at *p* < 0.05 using Pearson’s correlation coefficient. Relationships between soil and plants were explored by linear regression analyses. The coefficient of determination (R^2^) and mean square error (MSE) were used to assess goodness of fit. All analyses were performed with SPSS v. 9.1 (IBM, Armonk, NY, USA). SigmaPlot *v.* 14.5 software (Inpixon, Palo Alto, CA, USA) was used to plot the data.

## 3. Results and Discussion

### 3.1. Agrometeorological Conditions and Water Applied

During the experimental study, which comprised the summer and autumn seasons (DOY 150–300), the climate was typically Mediterranean with hot temperatures and irregular rainfall (normally below 250 mm), with an incidence of heavy storm events usually occurring during autumn–spring [50]. Figure S1 shows the daily values of air water potential (Ψ_air_), average air temperature (T_air_), reference crop evapotranspiration (ET_0_), and vapour pressure deficit (VPD). Ψ_air_ registered the highest day-to-day variability, producing daily maximum values in summer (DOY 228) of around −156 MPa (Figure S1). T_air_ and VPD showed similar behaviour, while ET_0_ decreased throughout the season. The highest absolute values of T (42.1 °C) and VPD (7.08 kPa) were reached in DOY 206, while the lowest values of the same climatic variables were observed in DOY 345 (T = 12.3 °C and VPD = 0.1 kPa), respectively (Figure S1). It is noteworthy that ET_0_ reached their maximum values one month earlier than VPD, with an accumulated value of 595 mm since the beginning of the study period (Figure S1).

Rain fell mainly in autumn, but occasionally in summer, with a total amount of 48.2 mm (Figure S2). Considering that irrigation was suspended in DOY 211–245, a total irrigation volume of 258.6 mm was applied to the nectarine trees during the experimental period, accounting for 56% of ET_0_ (Figures S1 and S2). As described in Vera et al. [3], the total annual requirements of well-irrigated, early maturing nectarine trees grown under Mediterranean conditions amounted to about 660 mm, with an irrigation frequency varying between 1 and 7 days per week, depending on the phenological period.

### 3.2. Soil-Based Water Status Indicators

Accurate determination of the soil water status (either Ψ_soil_ or Ɵ_v_) is not only important for irrigation management but is also a fundamental element in understanding the movement of soil water [51].

**Figure 2:**
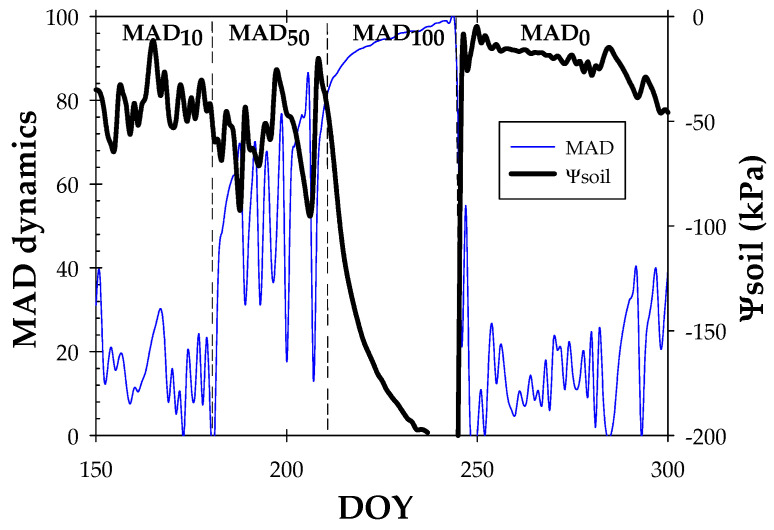
Seasonal trend of MAD dynamics (%) and average soil water potential (Ψ_soil_) at 0.30 and 0.60 m depths during the experimental period (DOY 150–300). The dashed vertical lines delimit the irrigation periods based on the MAD concept.

Figure 2 shows the seasonal trend of Ψ_soil_. The average values of Ψ_soil_ in each irrigation protocol were: −41.78 ± 2.28 kPa (MAD_10_), −55.02 ± 3.56 kPa (MAD_50_), −175.78 ± 5.19 kPa (MAD_100_), and −27.09 ± 0.91 (MAD_0_). When α increased, higher values of Ψ_soil_ reflected a higher soil water deficit situation, representing the relatively low availability of water retained in the soil profile for water uptake by plants [52].

In line with this, the Ɵ_v_ patterns at different soil depths are shown in Figure S3. In MAD_10_ and full irrigation (MAD_0_) periods, mean Ɵ_v_ values varied around field capacity values (29–32%) in the 0–0.50 m soil profile. This suggests that the process of soil water depletion by evapotranspiration and replenishment by irrigation or rainfall was more active in this soil layer [53]. There were no significant rainfall events during the experimental period that induced significant Ɵ_v_ increases even at a depth of 0.70 m (Figure S3). It is noteworthy that Ɵ_v_ variations were not only influenced by irrigation/rainfall events but also by diurnal climatic changes and root water uptake dynamics. This fact shows the sensitivity of capacitance probes to water variations in the soil and surrounding plant roots [44]. 

As expected, the minimum values (closer to the wilting point) of both Ψ_soil_ and Ɵ_v_ were reached at the end of the irrigation retention period (on DOY 245), with an average of Ψ_soil_ = −200 kPa and Ɵ_v_ =17%.

### 3.3. Plant-Based Water Status Indicators

**Figure 3:**
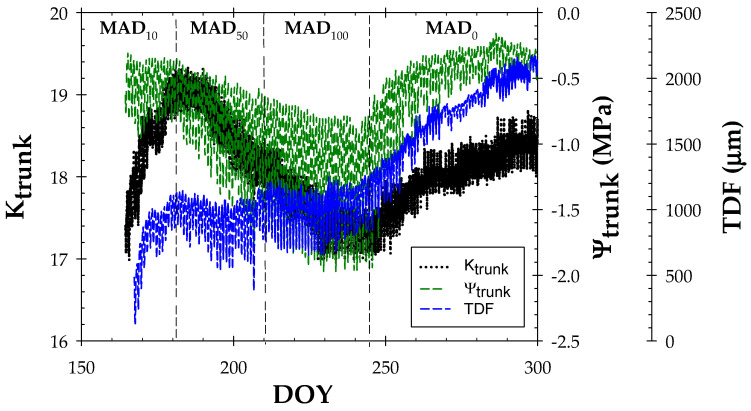
Seasonal trend of 15’ values of dielectric permittivity (K_trunk_), trunk water potential (Ψ_trunk_, MPa), and trunk diameter fluctuations (TDF, µm---) during the experiment (DOY 150–300). The dashed vertical lines delimit the irrigation periods based on the MAD concept.

The biosensors studied, especially minimally invasive ones such as MTs and TDR-305N, usually take at least a week to be well-adapted to the woody tissue, providing real values [46,47]. Once this aspect was achieved, continuous measurements of K_trunk_, Ψ_trunk_, and TDF monitored with TDR-305N, MTs, and LVDT sensors, respectively, varied according to the soil water deficit protocols as shown in Figure 3.

Both K_trunk_ and Ψ_trunk_ decreased during deficit periods, with this drop being more pronounced when the imposed soil deficit was higher. In MAD_10_, average values of K_trunk_/Ψ_trunk_ data were 18.62 ± 0.02/−0.42 ± 0.04 MPa, respectively. These values showed a huge decline in MAD_50_ and MAD_100_ periods, reaching the lowest values in DOY 242 (K_trunk_ = 17.3 and Ψ_trunk_ = −1.81 MPa). In pear trees, Blanco and Kalcits [31] reported a lower Ψ_trunk_ value of around −1.5 MPa, which agreed with the Ψ_stem_ values obtained with the pressure chamber. Those authors postulated that the feasibility of Ψ_trunk_ to continuously monitor plant water status after two consecutive growing seasons is highly dependent on the installation process of MTs, provided that good contact between the sensor and the xylem in the tree trunk is ensured to detect water flows.

Remarkably, although K_trunk_ mirrored the imposed soil deficit, its values fluctuated in a small range (barely 2 units), which is probably related to the distribution of xylem vessels [16].

Nectarine trees accumulated a trunk growth of up to 2000 µm at the end of the experiment, with a slowdown observed during the period of severe water deficit (MAD_100_) (Figure 3). In this regard, De la Rosa et al. [54] reported a reduction of about 45% in the trunk growth of nectarine trees subjected to regulated deficit irrigation (RDI) compared to fully irrigated trees.

Since TDF only refers to trunk growth, the derived index known as maximum daily shrinkage, MDS [39], has been shown to be more sensitive to changes in soil water content in early maturing *Prunus* sp. trees [40,54].

**Figure 4:**
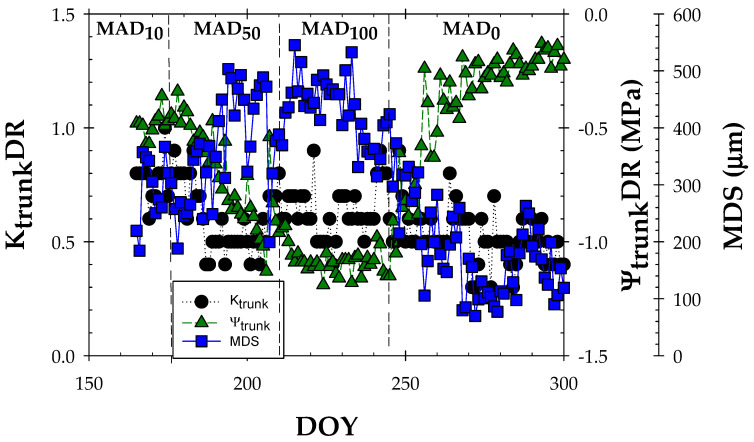
Seasonal evolution of maximum daily trunk shrinkage (MDS, µm), daily range dielectric permittivity (K_trunk_DR), and daily range trunk water (Ψ_trunk_DR, MPa) during the experimental period (DOY 150–300). The dashed vertical lines delimit the irrigation periods based on the MAD concept. Values are means of 4 replications.

Figure 4 shows the calculation of the daily range (DR) for K_trunk_ and Ψ_trunk_, using the same time-lapse as that of MDS measured with LVDT sensors. In MAD_10_, Ψ_trunk_DR, K_trunk_DR, and MDS showed averaged values of 3.01 ± 0.20, 0.39 ± 0.01, and 196.52 ± 9.52, respectively. Soil water deficit increased the MDS values, reaching a maximum value of 408 µm in DOY 206 (corresponding to moderate water deficit). In MAD_100_, MDS averaged 280.1 ± 7.49 µm, registering lower values than those at MAD_50_. Fernández and Cuevas [40] reviewed a decrease in the values of MDS in many fruit species when trees were subjected to a severe water deficit. This fact is one of the most limiting factors of this plant water status indicator, which is associated with a loss in woody tissue elasticity, as occurred in experimental nectarine trees in the MAD_100_ period (Figure 4).

**Figure 5:**
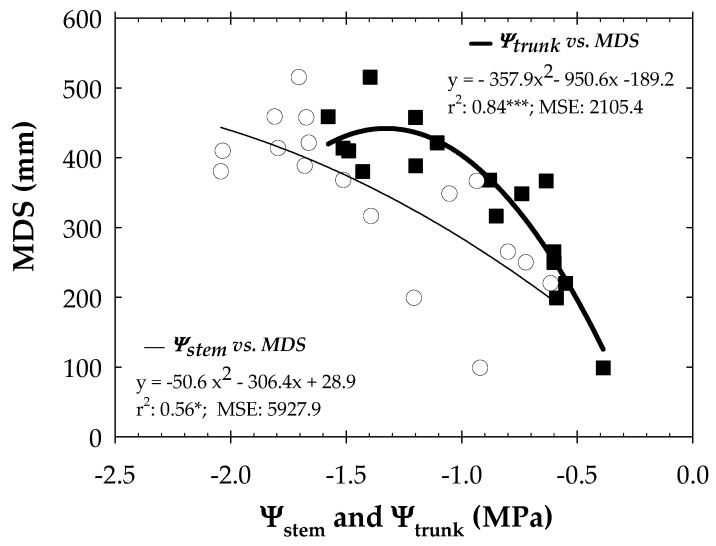
Relationship between mean diurnal values of stem water potential (Ψ_stem_ and MP: circles and thin line) and trunk water potential (Ψ_trunk_ and MPa: square and tick line) vs. maximum daily shrinkage (MDS). The values of MDS and Ψ_trunk_ represented are those calculated over the same time-lapse as for Ψ_stem_ during the experimental period. Values are means of 4 replications. *: *p* ≤ 0.05; ***: *p* ≤ 0.001. MSE: mean squared error.

Looking at the seasonal trends of both MDS and Ψ_trunk_DR, it is observed that Ψ_trunk_DR decreased (more negative) when MDS increased, reaching a minimum at Ψ_trunk_DR = −1.28 ± 0.06 MPa at the end of the MAD_100_ period (Figure 4). This behaviour is attributed to the fact that more stem water reserves would have been recruited to sustain leaf transpiration as the water deficit progressed [55,56]. Higher values of MDS corresponded to lower values of Ψ_stem_ up to − 1.5 MPa and Ψ_trunk_ of −1.2 MPa (Figure 5). A similar threshold value of Ψ_stem_ was reported by De la Rosa et al. [54] during postharvest of early maturing nectarine trees. Thereby, the gradient of 0.3 MPa between the values of Ψ_trunk_ monitored with MTs and Ψ_stem_ obtained with the pressure chamber agreed with this statement [15].

**Figure 6:**
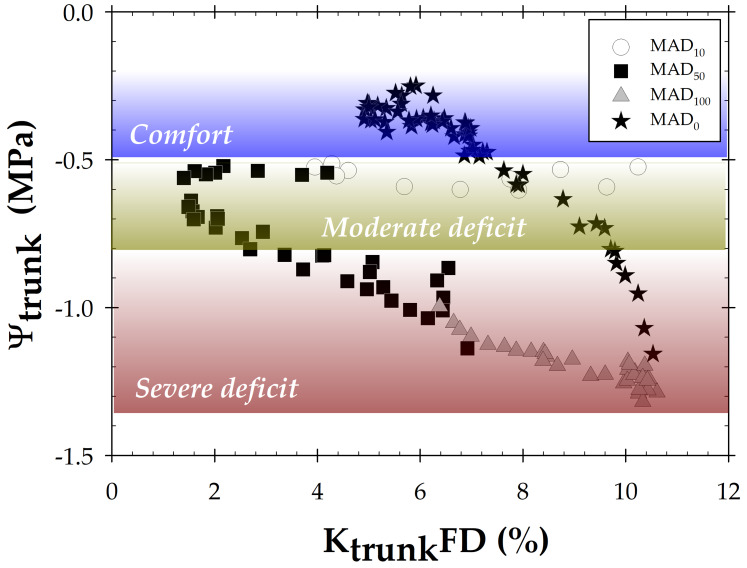
Relationship between depletion fraction of K_trunk_ (K_trunk_FD, %) and trunk water potential (Ψ_trunk_) for each experimental day. Values are means of 4 replications.

For its part, K_trunk_DR remains quite constant during the experiment (Figure 4), indicating the lower precision of this plant indicator for ascertaining plant water stress compared to the rest of the plant counterparts. Interestingly, when the fraction depletion of K_trunk_ (K_trunk_FD), calculated for each irrigation period, was correlated with Ψ_trunk_ values, a hysteresis phenomenon was exhibited (Figure 6), showing three different behaviours. The first one corresponded to the water comfort zone with a mean Ψ_trunk_ of −0.39 ± 0.01 MPa. Next to this, there was a moderate deficit zone, with values for the MAD_10_ and MAD_50_ periods averaging Ψ_trunk_ = −0.63 ± 0.01 MPa. It should be noted that the variability observed in K_trunk_FD was probably caused by the adaptation of the TDR-305N sensor to the woody trunk tissue [47]. The last zone corresponded to the period marked by a severe water deficit. In fact, 8 days after the beginning of the MAD_50_ irrigation period, the lower limit of the K_trunk_FD was reached, and, therefore, a sharp drop in the Ψ_trunk_ was observed until the beginning of the MAD_0_ period. Moreover, the seasonal hysteresis phenomenon indicated that irrigation recovery was not able to quickly and completely rehydrate the trunk vessels, as it coincided with foliar senescence, characteristic of this phenological period for nectarine trees [15] when full irrigation was applied.

When discrete measurements of Ψ_stem_ were correlated with real-time plant indicators, the best significant adjustment (r^2^ = 0.86 ***) was found between Ψ_stem_ vs. Ψ_trunk_, followed by Ψ_stem_ vs. MDS, and, to a lesser extent, between Ψ_stem_ vs. K_trunk_ (Table 1). Similar results were obtained when soil water variables were compared with real-time plant indicators, with the best fit between Ψ_trunk_ vs. Ɵ_v_ (r^2^ = 0.74 ***) and Ψ_soil_ (r^2^ = 0.71 ***) (Table 2). Measurements of Ψ_trunk_ involve the complete water pathway from the roots that absorb the available water in the soil to the stem, where water is transported through the xylem to the shoots, fruits and leaves, where it evaporates into the air [26]. Since MTs are able to directly monitor xylem water potential, determining the driving force for water transport between the bark and xylem vessels. This may explain why Ψ_trunk_ was more precise than MDS in assessing the water status of nectarine trees, and much more accurate than K_trunk_.

Some authors observed that MDS was a more suitable indicator than sap flow for irrigation scheduling in early maturing peach trees [56], and Remorini and Massai [55] reported that TDF measurements in peach trees were the first physiological indicator of variations in peach tree water functioning. In this sense, MDS showed a greater climatic dependence than Ψ_trunk_DR, with T_air_ being the climatic variable that best represented daily trunk variations (Table 3). De la Rosa et al. [54] claimed a greater dependence of MDS on climate when compared with Ψ_stem_. Our findings denoted that both Ψ_trunk_ and Ψ_stem_ clearly responded to the imposed MAD-based deficit, whereas MDS, which deals with daily trunk growth, was more influenced by the environmental conditions [40].

Comparatively, discrete determinations such as stem water potential at midday (Ψ_stem_) measured with the pressure chamber, and leaf gas exchange (P_n_, g_s_ and WUE_T_) also agreed with the soil water deficit protocols applied to nectarine trees (Figure S4). In MAD_10_ and full irrigation periods, Ψ_stem_ exhibited values in a range from −0.5 to −1.0 MPa, indicative of non-limiting soil water conditions. Similar results have been reported in other studies carried out on early maturing *Prunus* trees grown under Mediterranean conditions [14,21,54,56]. Stanley et al. [57] reported that leaf age can influence Ψ_stem_. Therefore, leaves at the beginning or at the end of the season might balance differently with the stem.

In MAD_10_, average values of P_n_, g_s_, and WUE_T_ were maximal (19.03 ± 1.06 μmol m^−2^ s^−1^, 320 ± 30.5 mmol m^−2^ s^−1^, and 4.09 ± 0.55 μmol mmol^−1^, respectively). An important effect of severe water limitations resulted in a decrease in P_n_ and g_s_, caused by a decrease in leaf expansion and stomatal closure among others [58], while WUE_T_ tended to increase as water deficit accumulated. Despite full irrigation leading to the recovery of Ψ_stem_ values at this stage, leaf gas exchange did not reach the values of the initial experiment, which can be explained by leaf senescence typical of deciduous fruit trees [59], which was more accelerated in early maturing cultivars such as the one used in this experiment. Moreover, the lower Ψ_stem_ values registered in MAD_100_ could have accelerated leaf senescence, which is a water-saving mechanism used to ensure plant survival. In addition, severe water deficits during the postharvest period had a negative impact on the mobilisation of root reserves necessary for the physiological processes in fruit trees [60].

**Figure 7:**
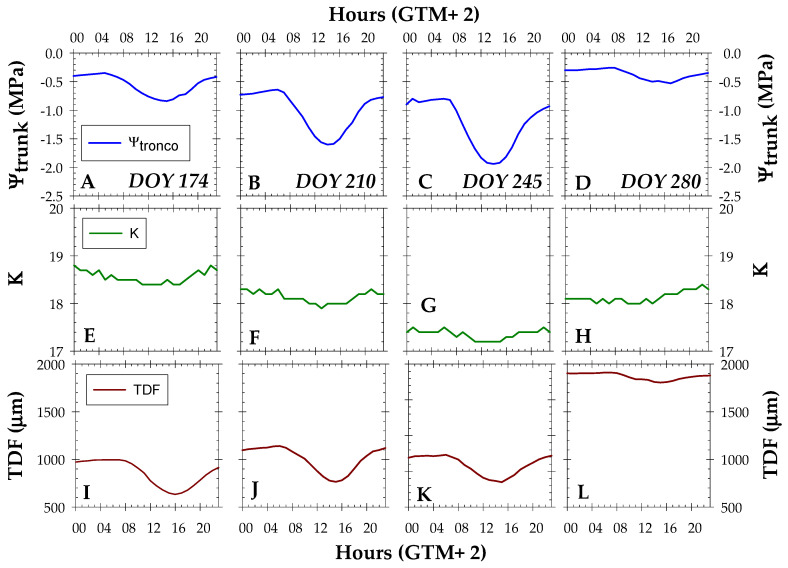
Daily dynamics of (**A**–**D**) trunk water potential (Ψ_stem_, MPa), (**E**–**H**) trunk dielectric permittivity (K_trunk_), and (**I**–**L**) trunk diameter fluctuations (TDF, µm) at the end of each irrigation period: MAD_10_ (light deficit, DOY 174), MAD_50_ (moderate deficit, DOY 210), MAD_100_ (severe deficit, no irrigation, DOY 245), and full irrigation (MAD_0_, DOY 280). Values are means of 4 replications.

Figure 7 analyses the diurnal rhythms of K_trunk_, Ψ_trunk_, and TDF measurements at the end of each irrigation protocol. Regarding seasonal patterns, the accumulated water deficit induced a significant decrease in Ψ_trunk_ and TDF but to a lesser extent in K_trunk_. For all irrigation conditions, minimum values of Ψ_trunk_ and K_trunk_ occurred at about 16:00 h GTM+2, while the minimum TDF was observed an hour later (17:00 h GTM+2). In this sense, changes in trunk diameter were delayed with respect to changes in Ψ_trunk_ in agreement with the results reported by Blanco and Kalcits [31]. In fact, continuous Ψ_trunk_ monitoring has recently demonstrated the ability to capture (re)hydration changes occurring within a day in response to environmental conditions better than other traditional indicators of plant water status [15,30].

The diurnal courses of Ψ_trunk_ and leaf gas exchange parameters also followed the established irrigation protocols (Figure S5). It should be noted that the most significant differences were found at midday for Ψ_stem_ and from sunrise to early morning for leaf gas exchange, coinciding with the period of maximum photosynthetic efficiency in all irrigation conditions studied.

### 3.4. Study of Plant Water Status Indicators Sensitivity

**Figure 8:**
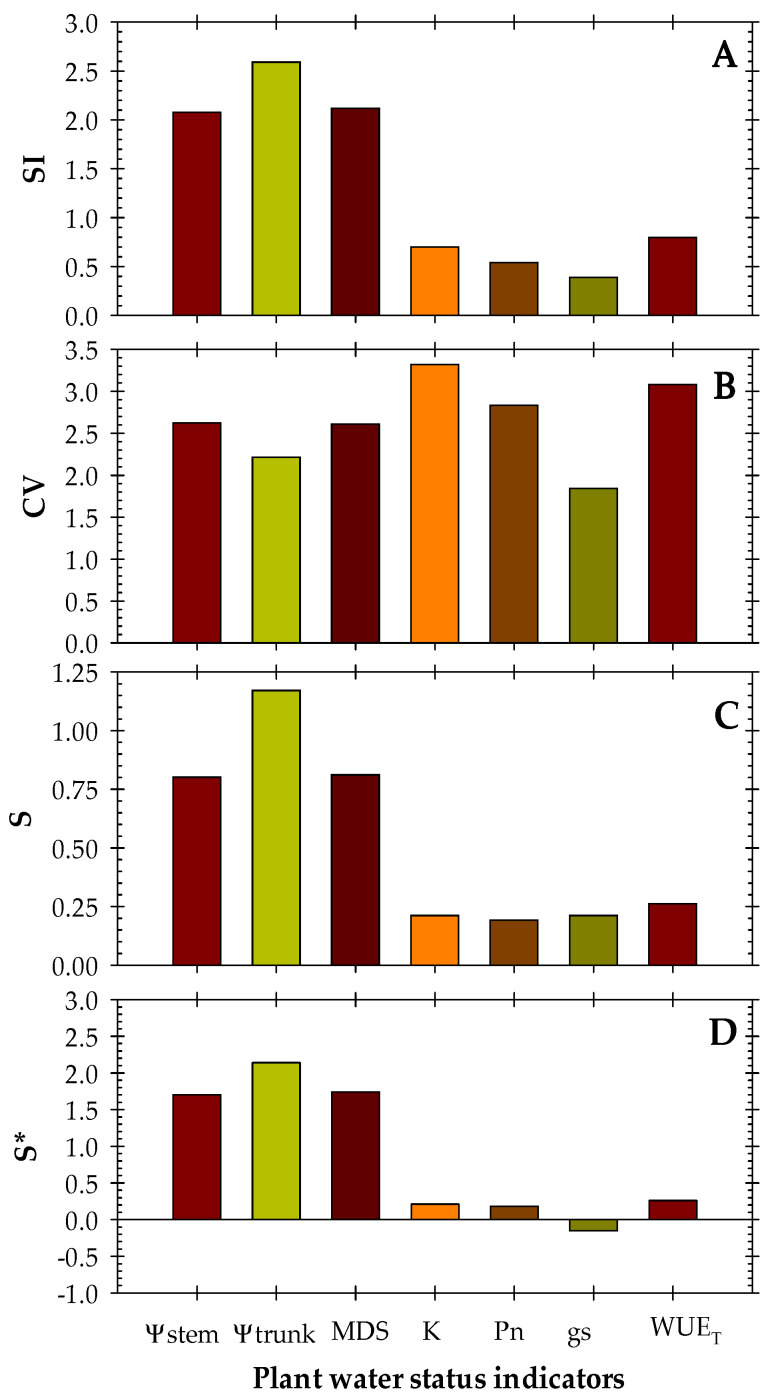
Sensitivity analysis (SI: signal intensity; CV: coefficient of variation; S: sensitivity (by Goldhamer and Fereres, [39]; and S*: corrected sensitivity by De la Rosa et al. [49]) for the indicators of plant water status during the experimental period.

Among the indexes derived from the plant biosensors studied, Ψ_trunk_, followed by MDS, registered the highest values of signal intensity (SI), with the value of MDS in the same range as that of Ψ_stem_ (Figure 8). Blanco and Kalcits [31] also detected Ψ_trunk_ as the most sensitive index for early detection of water stress in pear trees. However, K obtained the highest coefficient of variation, and, subsequently, the lowest S compared to the other continuous plant indicators. Comparing the sensitivity methods, the S* value [49] showed increasing values in the order Ψ_trunk_ > Ψ_stem_ > MDS. If continuous vs. discrete plant indicators are compared, Ψ_trunk_ reflected better performance in assessing nectarine tree water stress. In fact, one of the main issues with discrete measurements is the variability of values that can be caused by operator experience [61]. These findings emphasise the feasibility of real-time monitored Ψ_trunk_ measurements for assessing nectarine water status.

## 4. Conclusions

As a preliminary step for establishing digital irrigation based on plant measurements in early maturing nectarine trees, this study is the first assessment that jointly evaluates three different plant biosensors with automation capacity: MTs (microtensiometers), TDR-305N, and LVDT sensors (dendrometers) that continuously monitor nectarine water status in an attempt to replace the traditional pressure chamber method. Our results showed that although all these biosensors were able to monitor nectarine water status in real-time, the precision and sensitivity of the derived plant water status indicators (Ψ_trunk_, K_trunk_, and MDS) were highest for the MTs followed by the LVDT sensors and, to a lesser extent, the TDR embedded in the tree trunk. However, when K_trunk_ is calculated as a depletion fraction, its power improves. The strong correlation between Ψ_trunk_ vs. Ψ_stem_ and soil variables (Ɵ_v_ and Ψ_soil_), together with their dependence on the atmospheric demand and high sensitivity (although this was higher for MDS), confirmed the growing interest in using Ψ _trunk_ measured using less invasive trunk microtensiometer sensors (MTs). Since the accuracy of these sensors has not been validated so far, more research is needed to eventually use these biosensors for irrigation scheduling purposes. Our findings could contribute to the accuracy of digital farming in areas threatened by limited water availability for irrigation.

## Figures and Tables

**Table 1:** Coefficient of determination (r^2^) of lineal regressions between stem water potential (Ψ_stem_, kPa) vs. continuous plant indicator: maximum trunk shrinkage (MDS, µm), trunk dielectric permittivity (K_trunk_DR) and trunk water potential (Ψ_trunk_DR, MPa) monitored with LVDT, TDR-305N, and MTs biosensors, respectively.

Biosensor	Plant Indicator		Ψ_stem_ (MPa)
LVDT	MDS (µm)		0.55 *
TDR-305N	K_trunk_DR	vs.	0.12 ^ns^
MTs	Ψ_trunk_DR (MPa)		0.86 ***

*: *p* ≤ 0.05; ***: *p* ≤ 0.001; ns: not significant.

**Table 2:** Coefficient of determination (r^2^) of lineal regressions between soil water variables: soil water content (Ɵ_v_, %), and soil matric potential (Ψ_soil_, kPa) vs. continuous plant indicator: maximum trunk shrinkage (MDS, µm), trunk dielectric permittivity (K_trunk_DR), and trunk water potential (Ψ_trunk_DR, MPa), monitored using LVDT, TDR-305N, and MTs biosensors, respectively.

Biosensor	Plant Indicator		Ɵ_v_ (%)	Ψ_soil_ (kPa)
LVDT	MDS (µm)		0.647 **	0.473 *
TDR-305N	K_trunk_DR	vs.	0.114 ^ns^	0.064 ^ns^
MTs	Ψ_trunk_DR (MPa)		0.743 ***	0.718 ***

*: *p* ≤ 0.05; **: *p* ≤ 0.01; ***: *p* ≤ 0.001; ns: not significant.

**Table 3:** Coefficient of determination (r^2^) of lineal regressions between climatic variables air temperature (T_air_, °C), vapour pressure deficit (VPD), air water potential (Ψ_air_, MPa), and reference crop evapotranspiration (ET_0_, mm) vs. continuous plant indicator: maximum trunk shrinkage (MDS, µm), trunk dielectric permittivity (K_trunk_DR), and trunk water potential (Ψ_trunk_DR, MPa) monitored with LVDT, TDR-305N, and MTs biosensors, respectively.

Biosensor	Plant Indicator		T_air_ (°C)	VPD (kPa)	Ψ_air_ (MPa)	ET_0_ (mm)
LVDT	MDS (µm)		0.791 ***	0.594 ***	0.385 ^ns^	0.547 *
TDR-305N	K_trunk_DR	vs.	0.225 ^ns^	0.375 ^ns^	0.345 ^ns^	0.103 ^ns^
MTs	Ψ_trunk_DR (MPa)		0.719 ***	0.432 **	0.248 ^ns^	0.379 ^ns^

*: *p* ≤ 0.05; **: *p* ≤ 0.01; ***: *p* ≤ 0.001; ns: not significant.

## Data Availability

The data presented in this study are available upon request from the corresponding author.

## Supplementary Materials

The following supporting information can be downloaded at https://www.mdpi.com/article/10.3390/bios14120583/s1, Figures S1–S5. **Figure S1.** Seasonal evolution of daily air water potential (Ψair, MPa) (**A**), average air temperature (Tair) (**B**), reference evapotranspiration (ET_0_, mm day^−1^) (**C**), and vapour pressure deficit (VPD, kPa), (**D**) during the experimental period (DOY 150-300). The dashed vertical lines delimit the irrigation periods based on the MAD concept. **Figure S2.** Accumulated irrigation water applied and rainfall events during the experiment (DOY 150-300). The dashed vertical lines delimit the irrigation periods based on the MAD concept. **Figure S3.** Seasonal trend of soil water content (Ɵ_v_, %) at different depths: 0.10 m (**A**), 0.30 m (**B**), 0.50 m (**C**) and 0.70 m (**D**) during the experimental period (DOY 150-350). The dashed vertical lines delimit the irrigation periods based on the MAD concept. The dotted horizontal lines indicate the upper limit of field capacity (FC). Daily values correspond to means of 4 replications. **Figure S4.** Average values of midday stem water potential (Ψ_stem_, MPa) (**A**), net photosynthesis (P_n_, µmol m^−2^ s^−1^) (**B**), transpiration efficiency (WUE_T_, µmol mmol^−1^) (**C**), and stomatal conductance (g_s_, mmol m^−2^ s^−1^) (**D**) at each irrigation period: MAD_10_ (light deficit), MAD_50_ (moderate deficit), MAD_100_ (severe deficit, no irrigation) and full irrigation (MAD_0_). Values are means of 4 replications. means + SE. **Figure S5.** Daily dynamics (from 08:00 to 20-21:00 h GTM+2) of midday stem water potential (Ψ_stem_, MPa) (**A**–**D**), net photosynthesis (Pn, µmol m^−2^ s^−1^) (**E**–**H**), and stomatal conductance (gs, mmol m^−2^ s^−1^) (**I**–**L**), and transpiration efficiency (WUE_T_, µmol mmol^−1^) (**M**–**P**) at the end of each irrigation period: MAD_10_ (light deficit, DOY 174), MAD_50_ (moderate deficit, DOY 210), MAD_100_ (severe deficit, no irrigation, DOY 245) and full irrigation (MAD_0_, DOY 280). Each bar are means + SE of 4 leaves.

## References

[1] Fuentes-Peñailillo F., Gutter K., Vega R., Silva G.C. (2024). Transformative Technologies in Digital Agriculture: Leveraging Internet of Things, Remote Sensing, and Artificial Intelligence for Smart Crop Management. J. Sens. Actuator Netw..

[2] Carella A., Bulacio Fischer P.T., Massenti R., Lo Bianco R. (2024). Continuous Plant-Based and Remote Sensing for Determination of Fruit Tree Water Status. Horticulturae.

[3] Vera J., Conejero W., Conesa M.R., Ruiz-Sánchez M.C. (2019). Irrigation Factor approach based on soil water content: A nectarine orchard case study. Water.

[4] Xu C., Du Y., Zheng W., Liu Y., Li T. (2022). Data Image Aggregation Technology of Traffic Wireless Sensor Network. Mob. Inform. Syst..

[5] Martínez-Gimeno M.A., Jimenez-Bello M., Lidón A., Manzano J., Badal E., Pérez-Pérez J.G., Bonet L., Intrigliolo D.S., Esteban A. (2020). Mandarin irrigation scheduling by means of frequency domain reflectometry soil moisture monitoring. Agric. Water Manag..

[6] Vories E., Sudduth K. (2021). Determining sensor-based field capacity for irrigation scheduling. Agric. Water Manag..

[7] Casadesús J., Mata M., Marsal J., Girona J. (2012). A general algorithm for automated scheduling of drip irrigation in tree crops. Comp. Electron. Agric..

[8] Millán S., Casadesús J., Campillo C., Moñino M.J., Prieto M.H. (2019). Using soil moisture sensors for automated irrigation scheduling in a plum crop. Water.

[9] Domínguez-Niño J., Oliver-Manera J., Girona J., Casadesús J. (2020). Differential irrigation scheduling by an automated algorithm of water balance tuned by capacitance-type soil moisture sensors. Agric. Water Manag..

[10] Miller G.A., Farahani H.J., Hassell R.L., Khalilian A., Adelberg J.W., Wells C.E. (2014). Field evaluation and performance of capacitance probes for automated irrigation of watermelons. Agric. Water Manag..

[11] Osroosh Y., Peters R.T., Campbell C.S., Zhang Q. (2016). Comparison of irrigation automation algorithms for drip-irrigated apple trees. Comp. Electron. Agric..

[12] Merriam J.L. (1966). A management control concept for determining the economical depth and frequency of irrigation. Trans. ASAE.

[13] Allen R.G., Pereira J.S., Raes D., Smith M. (1998). Crop Evapotranspiration: Guidelines for Computing Crop Water Requirements.

[14] Conesa M.R., Conejero W., Vera J., Ramírez-Cuesta J.M., Ruiz-Sánchez M.C. (2019). Terrestrial and remote indexes to assess moderate deficit irrigation in early-maturing nectarine trees. Agronomy.

[15] Conesa M.R., Conejero W., Vera J., Ruiz-Sánchez M.C. (2023). Assessment of trunk microtensiometer as a novel biosensor to continuously monitor plant water status in nectarine trees. Front. Plant Sci..

[16] Conesa M.R., Vera J., Conejero W., Hernández-Santana V., Ruiz-Sánchez M.C. (2024). Trunk dielectric permittivity correlates with irrigation based on soil water content in fruit trees. Smart Agric. Technol..

[17] Noun G., Lo Cascio M., Spano D., Marras S., Sirca C. (2022). Plant-Based Methodologies and Approaches for Estimating Plant Water Status of Mediterranean Tree Species: A Semi-Systematic Review. Agronomy.

[18] Jones H.G. (2004). Irrigation scheduling: Advantages and pitfalls of plant-based methods. J. Exp. Bot..

[19] Fernández J.E. (2014). Plant-based sensing to monitor water stress: Applicability to commercial orchards. Agric. Water Manag..

[20] Naor A., Klein I., Doron I. (1995). Stem water potential and apple fruit size. J. Am. Soc. Hort. Sci..

[21] Abrisqueta I., Conejero W., Valdés-Vela M., Vera J., Ortuño M.F., Ruiz-Sánchez M.C. (2015). Stem water potential estimation of drip-irrigated early-maturing peach trees under Mediterranean conditions. Comp. Electron. Agric..

[22] Fernandes-Silva A., Oliveira M., Paço T.A., Ferreira I. (2019). Deficit Irrigation in Mediterranean Fruit Trees and Grapevines: Water Stress Indicators and Crop Responses. Irrigation in Agroecosystems.

[23] Shackel K.A., Ahmadi H., Biasi W., Buchner R., Goldhamer D., Gurusinghe S., Hasey J., Kester D., Krueger B., Lampinen B. (1997). Plant Water Status as an Index of Irrigation Need in Deciduous Fruit Trees. HortTechnol..

[24] Hueso A., González-García C., Atencia L.K., Novack J.C., Gómez del Campo M. (2023). Methodology of stem water potential measurement on hedgerow olive orchards. Span. J. Agric. Res..

[25] Pagay V., Santiago M., Sessoms D.A., Huber W.J., Vincent O., Pharkya A., Corso T.N., Lakso A.N., Stroock A.D. (2014). A microtensiometer capable of measuring water potential below −10 MPa. Lab Chip..

[26] Blanco V., Kalcsits L. (2021). Microtensiometers accurately measure stem water potential. Plants.

[27] Blanco V., Kalcsits L. (2024). Relating microtensiometer-based trunk water potential with sap flow, canopy temperature, and trunk and fruit diameter variations for irrigated ‘Honeycrisp’ apple. Front. Plant Sci..

[28] Steppe K., Vandegehuchte M.W., Tognetti R., Mencuccini M. (2015). Sap flow as a key trait in the understanding of plant hydraulic functioning. Tree Physiol..

[29] Pagay V. (2022). Evaluating a novel microtensiometer for continuous trunk water potential measurements in field-grown irrigated grapevines. Irrig. Sci..

[30] Christenson C.G., Gohardoust M.R., Calleja S.T., Thorp K.R., Tuller M., Pauli D. (2024). Monitoring cotton water status with microtensiometers. Irrig. Sci..

[31] Blanco V., Kalcsits L. (2023). Long-term validation of continuous measurements of trunk water potential and trunk diameter indicate different diurnal patterns for pear under water limitations. Agric. Water Manag..

[32] Gonzalez-Nieto L., Huber A., Gao R., Biasuz E.C., Cheng L., Stroock A.D., Lakso A.N., Robinson T.L. (2023). Trunk water potential measured with microtensiometers for managing water stress in “Gala” apple trees. Plants.

[33] Nadler A., Raveh E., Yermiyahu U., Green S. (2003). Evaluation of TDR use to monitor water content in stem of lemon trees and soil and their response to water stress. Soil Sci. Soc. Am. J..

[34] Nadler A., Raveh E., Yermiyahu U., Green S. (2006). Stress induced water content variations in mango stem by time domain reflectometry. Soil Sci. Soc. Am. J..

[35] Topp G., Annan J., Davis A. (1980). Electromagnetic determination of soil water content: Measurements in coaxial transmission lines. Water Res..

[36] He H., Turner N.C., Aogu K., Dyck M., Feng H., Si B., Wang J. (2021). Time and frequency domain reflectometry for the measurement of tree stem water content: A review, evaluation, and future perspectives. Agric. For. Meteorol..

[37] Holbrook N.M., Burns M., Sinclair T. (1992). Frequency and time-domain dielectric measurements of stem water content in the arborescent palm, *Sabal palmetto*. J. Exp. Bot..

[38] Hernandez-Santana V., Martínez-Fernández J., Moran C. (2008). Estimation of tree water stress from stem and soil water monitoring with time-domain reflectometry in two small forested basins in Spain. Hydrol. Process..

[39] Goldhamer D.A., Fereres E. (2001). Irrigation scheduling protocols using continuously recorded trunk diameter measurements. Irrig. Sci..

[40] Fernández J.E., Cuevas M.V. (2010). Irrigation scheduling from stem diameter variations: A review. Agric. For. Meteorol..

[41] Du S., Tong L., Zhang X., Kang S., Du S., Li S., Ding R. (2017). Signal intensity based on maximum daily stem shrinkage can reflect the water status of apple trees under alternate partial root-zone irrigation. Agric. Water Manag..

[42] Saxton K.E., Rawls W.J., Romberger J.S., Papendick R.I. (1986). Estimating generalized soil water characteristic from texture. Trans. ASAE.

[43] Nobel P. (1983). Biophysical Plant Physiology and Ecology.

[44] Evett S.R., Tolk J.A., Howell T.A. (2006). Soil Profile Water Content Determination: Sensor Accuracy, Axial Response, Calibration, Temperature Dependence, and Precision. Vadose Zone J..

[45] McCutchan H., Shackel K.A. (1992). Stem water potential as a sensitive indicator of water stress in prune trees (*Prunus domestica* L. cv. French). J. Am. Soc. Hort. Sci..

[46] Lakso A.M., Zhu A., Santiago M., Shackel V., Volkov A., Stroock A.D. (2022). A microtensiometer sensor to continuously monitor stem water potentials in woody plants-design and field testing. Acta Hortic..

[47] Schwartz R.C., Evett S.R., Anderson S., Anderson D. (2016). Evaluation of a direct-coupled TDR for determination of soil water content and bulk electrical conductivity. Vadose Zone J..

[48] Saito T., Yasuda H., Sakurai M., Acharya K., Sueki S., Inosako K., Yoda K., Fujimaki H., Abd Elbasit M.A.M., Eldoma A.M. (2016). Monitoring of stem water content of native and invasive trees in arid environments using gs3 soil moisture sensors. Vadose Zone J..

[49] De la Rosa J.M., Conesa M.R., Domingo R., Pérez-Pastor A. (2014). A new approach to ascertain the sensitivity to water stress of different plant water indicators in extra-early nectarine trees. Sci. Hortic..

[50] Paxian A., Hertig E., Seubert S., Vogt G., Jacobeit J., Paeth H. (2015). Present-day and future Mediterranean precipitation extremes asessed by different statistical approaches. Clim. Dyn..

[51] Jones H.G. (2007). Monitoring plant and soil water status: Established and novel methods revisited and their relevance to studies of drought tolerance. J. Exp. Bot..

[52] Thompson R.B., Gallardo M., Aguilera T., Valdez L.C., Fernández M.D. (2006). Evaluation of watermark sensor for use with drip irrigated vegetable crops. Irrig. Sci..

[53] Wang F., Hu W., Li T. (2009). The influence of freeze–thaw cycles of active soil layer on surface runoff in a permafrost watershed. J. Hidrol..

[54] De la Rosa J.M., Domingo R., Gómez-Montiel J., Pérez-Pastor A. (2015). Implementing deficit irrigation scheduling through plant water stress indicators in early nectarine trees. Agric. Water Manag..

[55] Wong S., Cowan I., Farquhar G. (1979). Stomatal conductance correlates with photosynthetic capacity. Nature.

[56] Remorini D., Massai R. (2003). Comparison of water status indicators for young peach trees. Irrig. Sci..

[57] Conejero W., Alarcón J.J., García-Orellana Y., Abrisqueta J.M., Torrecillas A. (2007). Daily sap flow and maximum daily trunk shrinkage measurements for diagnosing water stress in early maturing peach trees during the post-harvest period. Tree Physiol..

[58] Stanley C.D., Harbaugh B.K., Price J.F. (1983). Environmental factors influencing leaf water potential of chrysanthemum. J. Am. Soc. Hortic. Sci..

[59] Bertold M., Dox I., De Boeck H., Willems P., Leys S., Papadimitriou D., Campioli M. (2021). Does drought advance the onset of autumn leaf senescence in temperate deciduous forest trees?. Biogeosciences.

[60] Conesa M.R., Conejero W., Vera J., Ruiz-Sánchez M.C. (2022). Root Reserves Ascertain Postharvest Sensitivity to Water Deficit of Nectarine Trees. Agronomy.

[61] Levin A.D. (2019). Re-evaluating pressure chamber methods of water status determination in field-grown grapevine (*Vitis* spp.). Agric. Water Manag..

