# Supplementary material for: Plant Biosensors Analysis for Monitoring Nectarine Water Status
Source: Biosensors (Basel). 2024 Nov 30;14(12):583. doi: 10.3390/bios14120583 (PMC11674555; doi:10.3390/bios14120583)
Supplement: Supplementary file 1 [file biosensors-14-00583-s001.zip › biosensors-3273192-supplementary.pdf]

biosensors

MDPI

Supplementary Material

Plant biosensors analysis for monitoring nectarine water status

María R. Conesa, Wenceslao Conejero, Juan Vera and M. Carmen Ruiz Sánchez

Irrigation Department, Centro de Edafología y Biología Aplicada del Segura (CEBAS-CSIC), P.O. Box 164, 30100 Murcia, Spain; wenceslao@cebas.csic.es (W.C.); jvera@cebas.csic.es (J.V.); mcruiz@ceba.csic.es (M.C.R-S)
* Correspondence: mrconesa@cebas.csic.es (M.R.C); Tel.: +34-968-396200

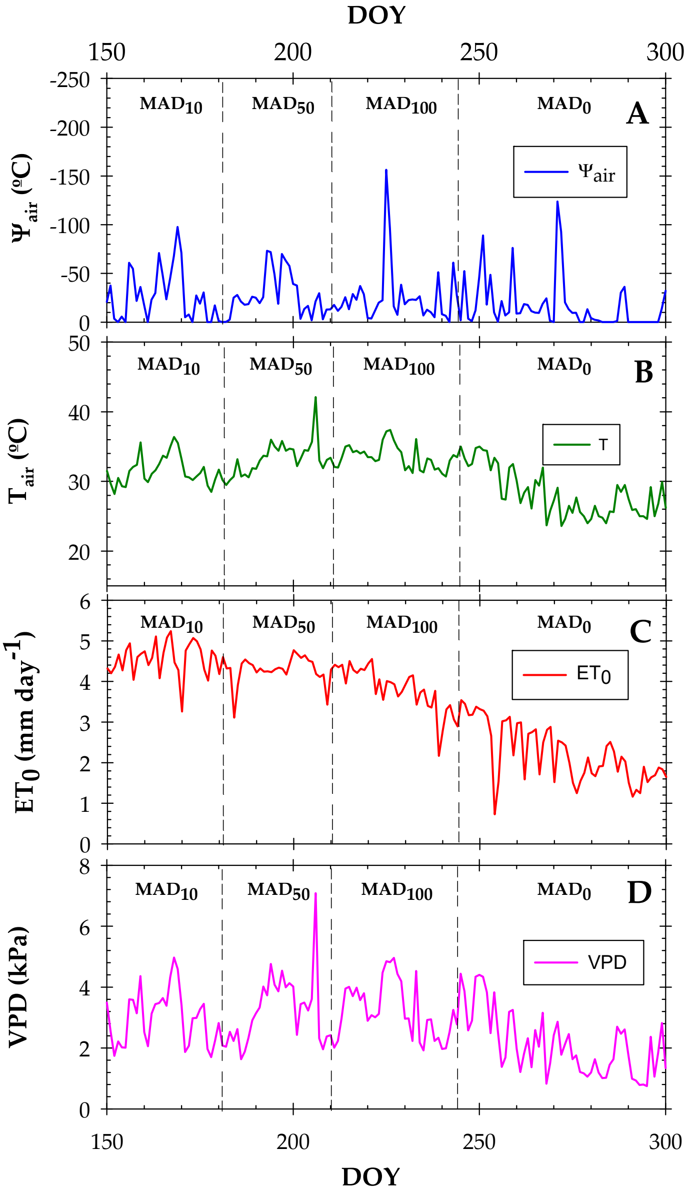

Figure S1. Seasonal evolution of daily air water potential (Ψair, MPa) **(A),** average air temperature (Tair) **(B),** reference evapotranspiration (ET_0, mm day^{-1}) **(C),** and vapour pressure deficit (VPD, kPa), **(D)** during the experimental period (DOY 150-300). The dashed vertical lines delimit the irrigation periods based on the MAD concept.

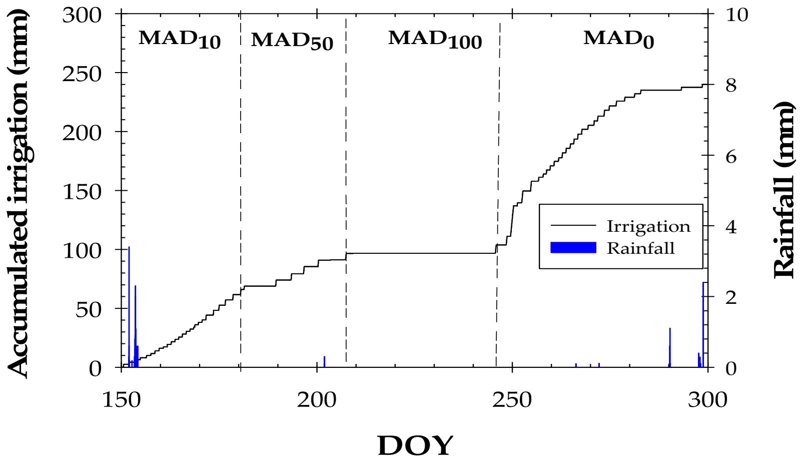

Figure S2. Accumulated irrigation water applied and rainfall events during the experiment (DOY 150-300). The dashed vertical lines delimit the irrigation periods based on the MAD concept.

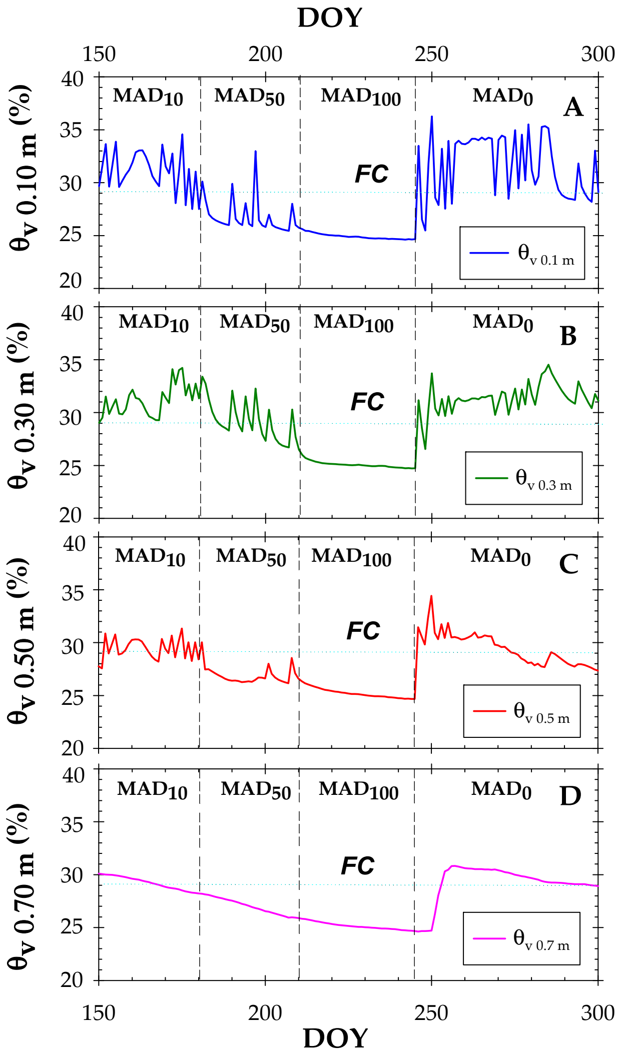

Figure S3. Seasonal trend of soil water content (θ_v, %) at different depths: 0.10 m **(A),** 0.30 m **(B),** 0.50 m **(C)** and 0.70 m **(D)** during the experimental period (DOY 150-350). The dashed vertical lines delimit the irrigation periods based on the MAD concept. The dotted horizontal lines indicate the upper limit of field capacity (FC). Daily values correspond to means of 4 replications.

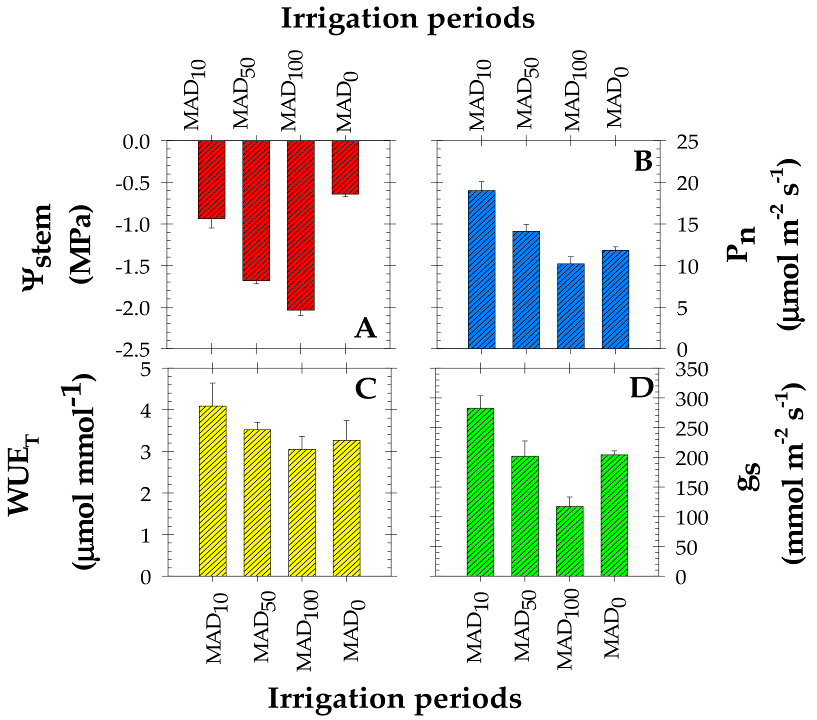

Figure S4. Average values of midday stem water potential (Ψ_{stem}, MPa) **(A),** net photosynthesis (P_n, µmol m^{-2} s^{-1}) (B), transpiration efficiency (WUE_T, µmol $mmol^{-1}$) **(C),** and stomatal conductance (g_s, mmol m^{-2} s^{-1}) **(D)** at each irrigation period: MAD_{10} (light deficit), MAD_{50} (moderate deficit), MAD_{100} (severe deficit, no irrigation) and full irrigation (MAD_0). Values are means of 4 replications. means + SE.

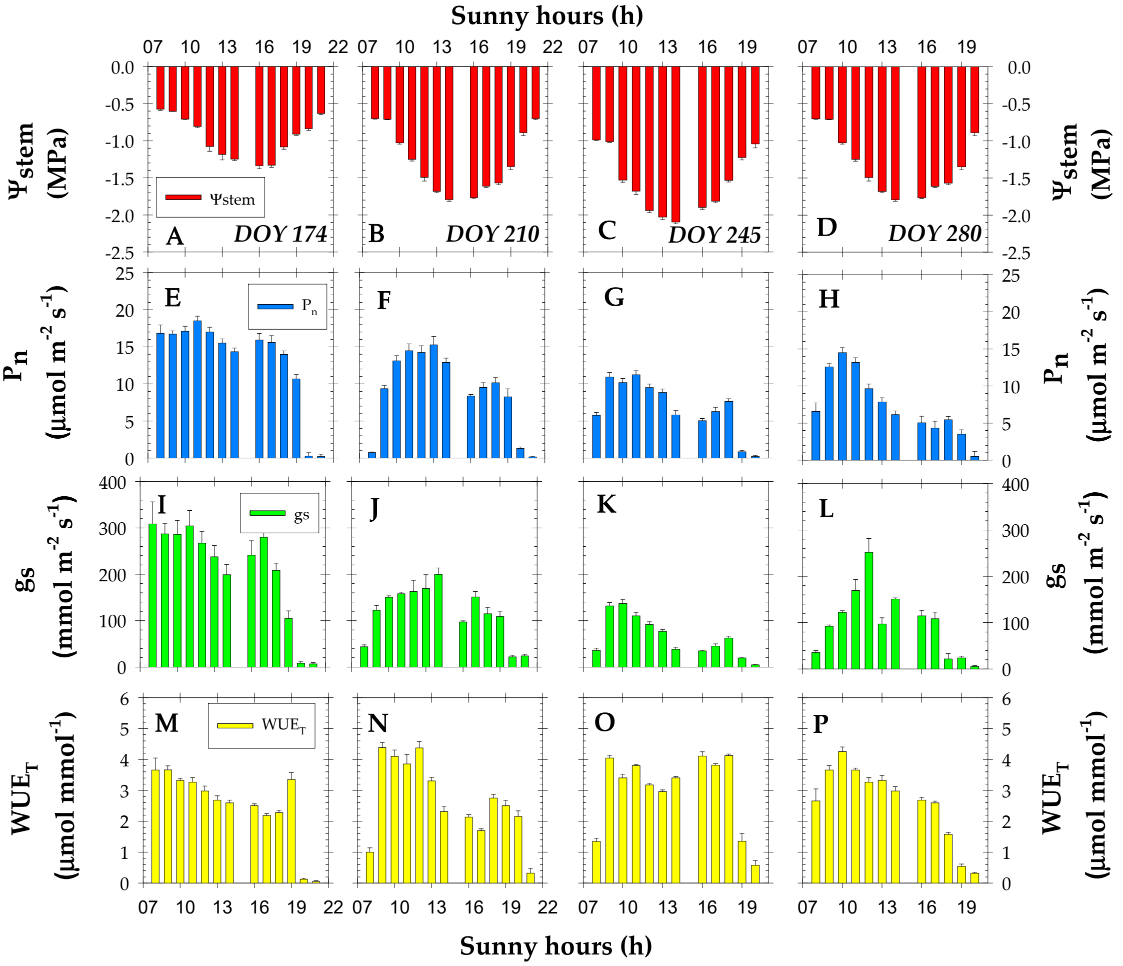

Figure S5. Daily dynamics (from 08:00 to 20-21:00 h GTM+2) of midday stem water potential (Ψ_{stem}, MPa) **(A-D)**, net photosynthesis (Pn, µmol m^{-2} s^{-1}) **(E-H)**, and stomatal conductance (gs, mmol m^{-2} s^{-1}) **(I-L),** and transpiration efficiency (WUE_T, µmol mmol^{-1}) **(M-P)** at the end of each irrigation period: MAD_{10} (light deficit, DOY 174), MAD_{50} (moderate deficit, DOY 210), MAD_{100} (severe deficit, no irrigation, DOY 245) and full irrigation (MAD_0, DOY 280). Each bar are means + SE of 4 leaves.
